# Treatment for Pulmonary Vein Stenosis After Atrial Fibrillation Ablation: The Potential of Novel Surgical Reconstruction

**DOI:** 10.1016/j.jaccas.2026.108603

**Published:** 2026-07-29

**Authors:** Seigo Yamashita

**Affiliations:** Division of Cardiology, The Jikei University Katsushika Medical Center, Tokyo, Japan

**Keywords:** atrial fibrillation, ablation, complication

**Figure:**
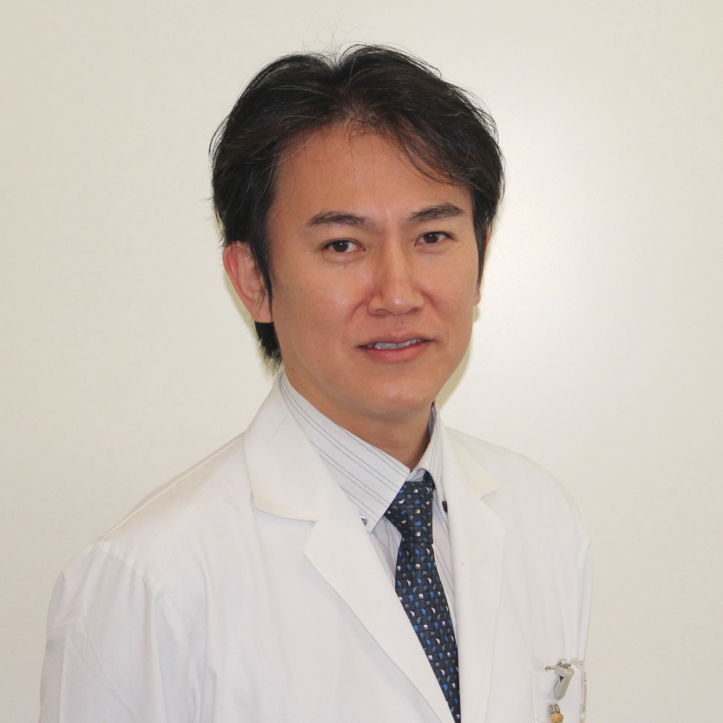

Pulmonary vein (PV) stenosis/occlusion occurring after catheter ablation (PV isolation) for atrial fibrillation can occasionally become a life-threatening condition and must be recognized as a significant complication that cannot be overlooked. It has been previously reported to occur not only with radiofrequency ablation^1^ but also after the use of single-shot devices such as the cryoballoon,^2^ hot balloon,^3^ and laser balloon.^4^ However, a problem remains that no definitive treatment for PV stenosis/occlusion has been established yet. Regarding the efficacy of PV angioplasty using a transvenous catheter, a meta-analysis has reported that stent placement is associated with a significantly lower restenosis rate compared with balloon angioplasty alone. Nevertheless, restenosis still occurs in approximately 22% of cases after stenting, indicating that this approach is not a complete solution.^5^ In addition, no dedicated stent for PV placement has been developed, and stents designed for peripheral arteries are used off-label. This leads to the significant issue of size mismatch, particularly regarding stent length. Although the precise mechanism of in-stent restenosis remains unclear, observations using nonobstructive general angioscopy suggested neointimal changes rather than thrombus formation.^6^ Therefore, restenosis is presumed to be mainly due to inflammation and intimal hyperplasia caused by stent exposure, rather than poor anticoagulation. Consequently, using a larger stent size may be expected to reduce the restenosis rate; however, in cases of occlusion, the distal PV is often shrunken, and actual dilation to a larger size carries a risk of rupture. Furthermore, placing a large stent confined to the stenotic lesion at the PV ostium is practically difficult in many cases. In a previous report, drug-eluting stents (diameter: 3-5 mm) have been associated with a higher restenosis rate compared with large-diameter bare metal stent (diameter: 7-10 mm).^7^ Although some case reports have demonstrated the effectiveness of drug-coated balloon (DCB) angioplasty, the maximum balloon diameter of available DCBs is often limited (≤7 mm), frequently resulting in insufficient dilation. Moreover, the PV walls have a thinner tunica media layer relative to arterial walls, increasing the risk of dissection and reocclusion with angioplasty alone.^8^ Therefore, it remains unclear whether DCB can altogether replace stenting.

On the other hand, several surgical treatments for PV stenosis/occlusion have been reported, indicating patch angioplasty using bovine pericardium or polytetrafluorethylene patches,^9^ and the sutureless technique, which involves covering the stenosis with the left atrial appendage (LAA).^10^ However, the former had a high restenosis rate of 39% over 5 years, whereas the latter has been limited to sporadic case reports without large-scale series. Given that there are rare procedures and few surgeons have extensive experience with them, an established method for complete revascularization has yet to be determined at this time. In this issue of *JACC: Case Reports*, Umesue et al^11^ reported a novel technique that bypasses the stenotic lesion by anastomosing the distal portion of the PV directly to the LAA, thereby restoring pulmonary blood flow to the left atrium. Since this technique does not involve manipulating the diseased lesion but instead creates an anastomosis between the normal distal vessel tissue of the left superior PV and the LAA, it may reduce the risk of restenosis. In fact, computed tomography (CT) performed 1 week after the surgery showed no evidence of significant restenosis, and midterm follow-up using pulmonary perfusion scintigraphy demonstrated that improvement in pulmonary blood flow was maintained at 6 months. Although this technique is limited to the left superior PV due to the anastomosis with the LAA, it remains a promising approach given that this vessel is frequently affected by PV stenosis. In addition, because this technique does not use stent or artificial patch, it offers the advantage of not requiring antithrombotic therapy. However, in this case report, follow-up examinations including pulmonary perfusion scintigraphy and CT were performed at only a single time point postoperatively. Because the scintigraphy did not demonstrate complete restoration of blood flow, it remains unclear whether the improvement in pulmonary blood flow has been sustained postoperatively. Moreover, because luminal evaluation was not demonstrated on the CT image in the figure, the possibility of mild restenosis at the anastomotic site cannot be completely ruled out. These questions could be addressed through serial follow-up examinations, so it appears important to carefully monitor the temporal changes in the future. Furthermore, as this is a single case report, accumulation of additional cases will be necessary to demonstrate the efficacy of this technique.

Finally, although the advent of the pulsed-field ablation era appears to have nearly eliminated the risk of PV stenosis/occlusion, there has been a case report of progression to severe stenosis following the use of pulsed-field ablation during a repeat ablation procedure.^12^ Moreover, given that radiofrequency ablation remains highly convenient and versatile and is expected to continue being used in the future, treatment strategies for PV stenosis/occlusion should remain a topic of thorough discussion going forward.

## Funding Support and Author Disclosures

The author has reported that he has no relationships relevant to the contents of this paper to disclose.
